# Group-13 and group-15 doping of germanane

**DOI:** 10.3762/bjnano.8.164

**Published:** 2017-08-09

**Authors:** Nicholas D Cultrara, Maxx Q Arguilla, Shishi Jiang, Chuanchuan Sun, Michael R Scudder, R Dominic Ross, Joshua E Goldberger

**Affiliations:** 1 Department of Chemistry and Biochemistry, Ohio State University, Columbus, Ohio, 43210-1340, United States of America

**Keywords:** doping, electronic behavior, germanane, two-dimensional materials

## Abstract

Germanane, a hydrogen-terminated graphane analogue of germanium has generated interest as a potential 2D electronic material. However, the incorporation and retention of extrinsic dopant atoms in the lattice, to tune the electronic properties, remains a significant challenge. Here, we show that the group-13 element Ga and the group-15 element As, can be successfully doped into a precursor CaGe_2_ phase, and remain intact in the lattice after the topotactic deintercalation, using HCl, to form GeH. After deintercalation, a maximum of 1.1% As and 2.3% Ga can be substituted into the germanium lattice. Electronic transport properties of single flakes show that incorporation of dopants leads to a reduction of resistance of more than three orders of magnitude in H_2_O-containing atmosphere after As doping. After doping with Ga, the reduction is more than six orders of magnitude, but with significant hysteretic behavior, indicative of water-activation of dopants on the surface. Only Ga-doped germanane remains activated under vacuum, and also exhibits minimal hysteretic behavior while the sheet resistance is reduced by more than four orders of magnitude. These Ga- and As-doped germanane materials start to oxidize after one to four days in ambient atmosphere. Overall, this work demonstrates that extrinsic doping with Ga is a viable pathway towards accessing stable electronic behavior in graphane analogues of germanium.

## Introduction

Since the discovery of graphene [1], the quest to discover and measure novel two dimensional and layered materials has led to the investigation of group-14 and group-15 allotropes of graphene and graphane [1–14], transition-metal dichalcogenides [15–19], and layered van der Waals materials [20–22]. Germanane, a hydrogen-terminated graphane analogue of germanium, has garnered considerable attention in the field of 2D materials on account of its direct band gap [5,23–24], large predicted electron mobility, and the ability to controllably tune the optoelectronic properties via covalent modification with surface ligands [3,24–28]. While the electron mobility of germanane at room temperature has been predicted to be greater than 18,000 cm^2^·V^−1^·s^−1^, transport measurements on non-extrinsically doped crystals were highly resistive, indicating the need of extrinsic dopants to access devices with lower resistivity. In previous studies, the resistivity of germanane was reduced through the incorporation of phosphorus only when activated in the presence of atmospheric water [29]. Recently [30], undoped germanane field-effect transistors were reported with device hole mobilites ranging from 70 to 150 cm^2^·V^−1^·s^−1^ from room temperature to low temperature, indicating that germanane has the potential to be a viable electronic building block for 2D transistors. Together, this emphasizes the need for further control of doping behavior in these materials.

The preparation of GeH requires the synthesis of a CaGe_2_ precursor phase followed by its topotactic deintercalation in HCl. GeH is a metastable phase, and begins to amorphize when annealed above 75 °C. Consequently, traditional doping processes such as the direct ion-implantation of GeH cannot be used as they require a high-temperature for post annealing to heal the lattice. Due to the existence of a large number of closely related layered Zintl phases with group-13 and group-15 elements that are structurally similar to CaGe_2_ dopant elements can be partially substituted into the germanium lattice of the CaGe_2_ precursor [29,31]. Providing that these elements are retained in the germanium framework after the topotactic deintercalation process, the effect on the electronic transport behavior of GeH should be appreciable. Having previously grown phosphorus-doped GeH (P:GeH) [29] using this method, here, we explored whether other group-13 and group-15 elements (Al, Ga, As and Sb) can be included as dopants onto the germanane framework, and how these dopants affect the stability and electronic properties of GeH.

Herein, we show that Ga and As can be doped into the CaGe_2_ precursor phase and are retained on the germanane lattice after topotactic deintercalation. Using X-ray fluorescence (XRF) and X-ray photoelectron spectroscopy (XPS) we show that up to 1.1% and 2.3% of As and Ga, respectively, can be substituted onto the germanane lattice. In contrast to pristine GeH, these materials begin to oxidize after 24 to 96 hours in ambient atmosphere. In both cases, the incorporation of more dopant produced lower sheet resistances in H_2_O-containing ambient atmosphere, while only the gallium-doped samples continue to show dopant activation under vacuum and H_2_O-free conditions.

## Results and Discussion

**Figure 1:**
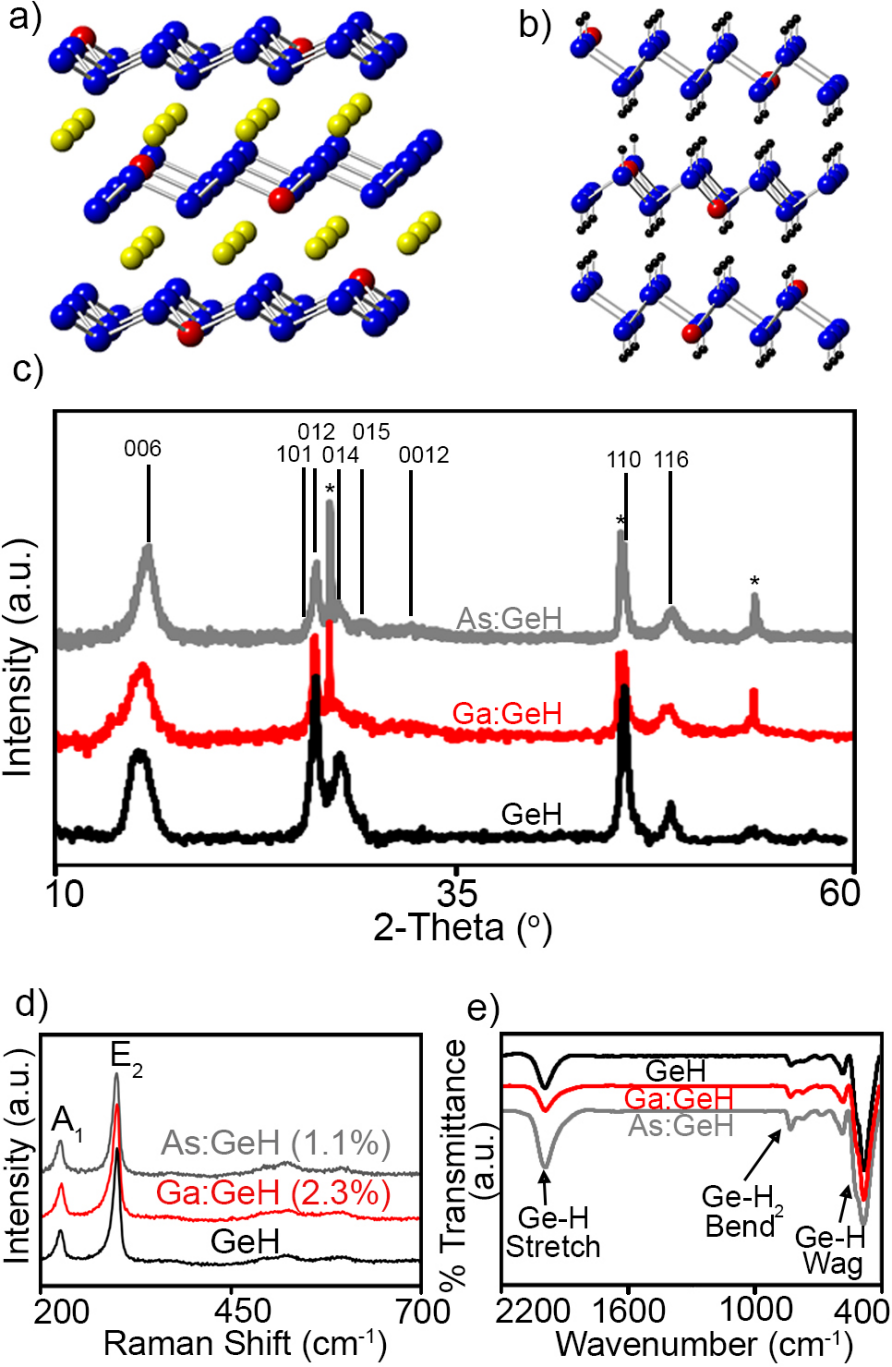
Schematic diagram of doped a) CaGe_2_ and b) GeH after detintercalation. Red represents the dopant atom, blue is germanium, yellow is calcium and black is hydrogen. The number of dopants depicted here is purposefully inflated for visual effect. c) Powder XRD, d) Raman spectra, and e) FTIR spectra of GeH (black), 2.3% Ga:GeH (red), and 1.1% As:GeH (gray). The starred peaks in the XRD show residual germanium.

First, we explored whether crystals of CaGe_2_ doped with Al, Ga, As, and Sb at 0.1% could be synthesized (Figure 1a). Of these dopants only Al, Ga, and As were successfully incorporated into the CaGe_2_ framework. After topotactic deintercalation in HCl, GeH platelets doped with Ga and As were successfully obtained (Figure 1b), while the Al-doped CaGe_2_ crystals disintegrated into small micrometer-sized particles not suitable for bulk transport measurements. Subsequently, we tried to synthesize Ga- and As-doped CaGe_2_ with 0.1–9% atomic substitutions. However, CaGe_2_ crystals only formed with less than 3% of added dopant. Figure 1c shows the powder X-ray diffraction (XRD) pattern of undoped GeH reported by Bianco et al. [25], and those of the highest doped Ga:GeH and As:GeH samples. All the deintercalated phases can be indexed [32] to a 6-layer rhombohedral unit cell with lattice parameters *a* = 3.97 Å and *c* = 33.22 Å. Neither a significant difference between the phases nor other peaks indicative of impurity phases are observed. The peaks labeled with asterisks show residual germanium in the sample. The Raman spectra (Figure 1d) of these doped crystals all exhibit A_1_ (out-of-plane) and E_2_ (in-plane) modes at 228 cm^−1^ and 302 cm^−1^, respectively, with no change in peak location, shape or A_1_/E_2_ intensity ratio compared to undoped GeH. Fourier transform infrared spectra of these samples (FTIR) also further show clear spectroscopic signatures for the formation of GeH. An extremely strong Ge–H stretching mode is observed at about 2000 cm^−1^ as well as characteristic wagging modes at 570, 507 and 475 cm^−1^, and the edge/defect Ge–H_2_ defect modes at 770 and 820 cm^−1^. No additional features indicative of As–H or Ga–H were observed, because the small concentration of Ga and As makes it impossible to completely determine the actual chemical environment of these dopants using a bulk technique such as IR spectroscopy.

**Figure 2:**
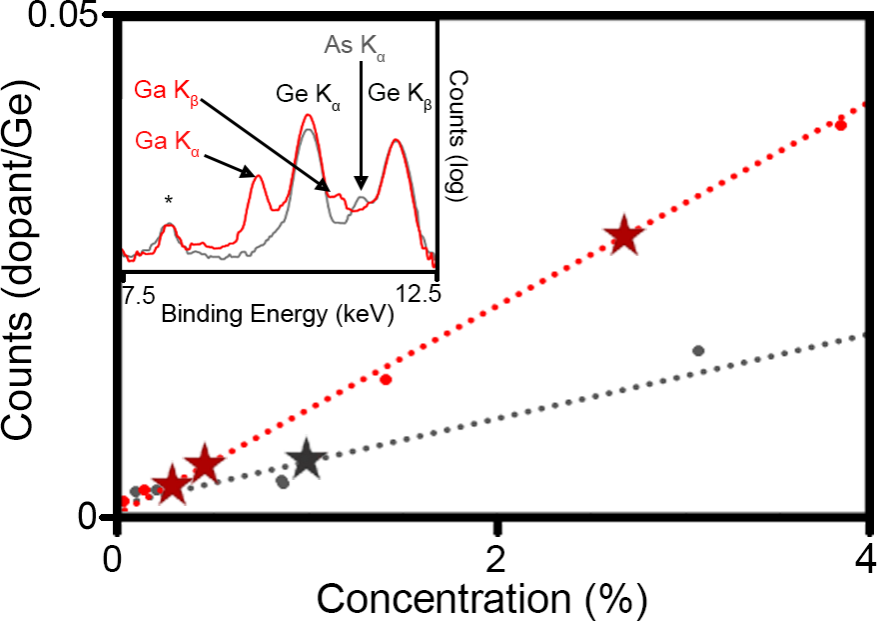
XRF calibration curve of Ga:GeH (red) and As:GeH (gray). The stars represent measured concentrations in the samples. Inset shows XRF spectra of the Ga:GeH (red) and As:GeH (gray) samples with the highest doping content. The asterisk denotes a silicon escape peak from the major Ge K_α_ peak.

The retention and concentration of Ga and As dopants in the lattice was determined for each system using XRF (Figure 2). A calibration curve using the ratio of Ga/As Kα to Ge Kα was prepared with standards of elemental Ge and As or of Ga_2_O_3_. The XRF measurements showed that the highest concentration of Ga in Ga:GeH to be 2.3%, and the highest concentration of As in As:GeH was 1.1%. XRF analysis of GeH synthesized with greater than 1% As substitution, always yielded a ratio As/Ge of ca. 1.1% in GeH indicating that this is the maximum amount of As that can be substituted in CaGe_2_. The lack of any other distinguishing phase in the XRD suggests that Ga and As are part of the germanane lattice.

**Figure 3:**
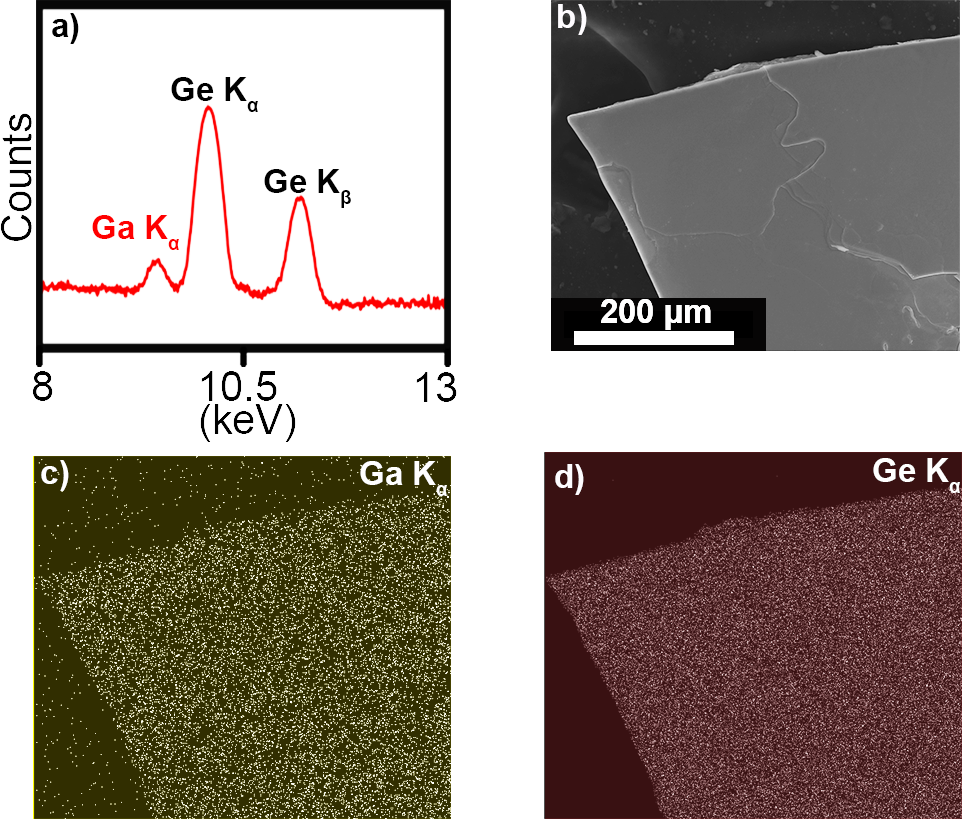
a) EDX spectrum of the 2.3% Ga:GeH platelet shown in the SEM image in panel b). EDX maps of c) gallium (yellow) and d) germanium (red) show retention of the dopants in the germanane crystal as well as their uniform distribution.

Scanning electron microscopy (SEM) with energy-dispersive X-ray spectroscopy (EDX) provided further verification of the incorporation of dopant atoms into GeH. As a representative example, the EDX spectrum of a single 2.3% Ga:GeH platelet shows the presence of both Ge K and Ga K peaks (Figure 3a). Figure 3b–d shows an SEM image, and the maps of Ga K_α_ signal and Ge K_α_ signal, of a corner of a Ga:GeH platelet. These EDX maps show that there is a uniform distribution of gallium and germanium throughout the germanane crystal. This confirms the retention of the Ga dopant into the germanane lattice.

**Figure 4:**
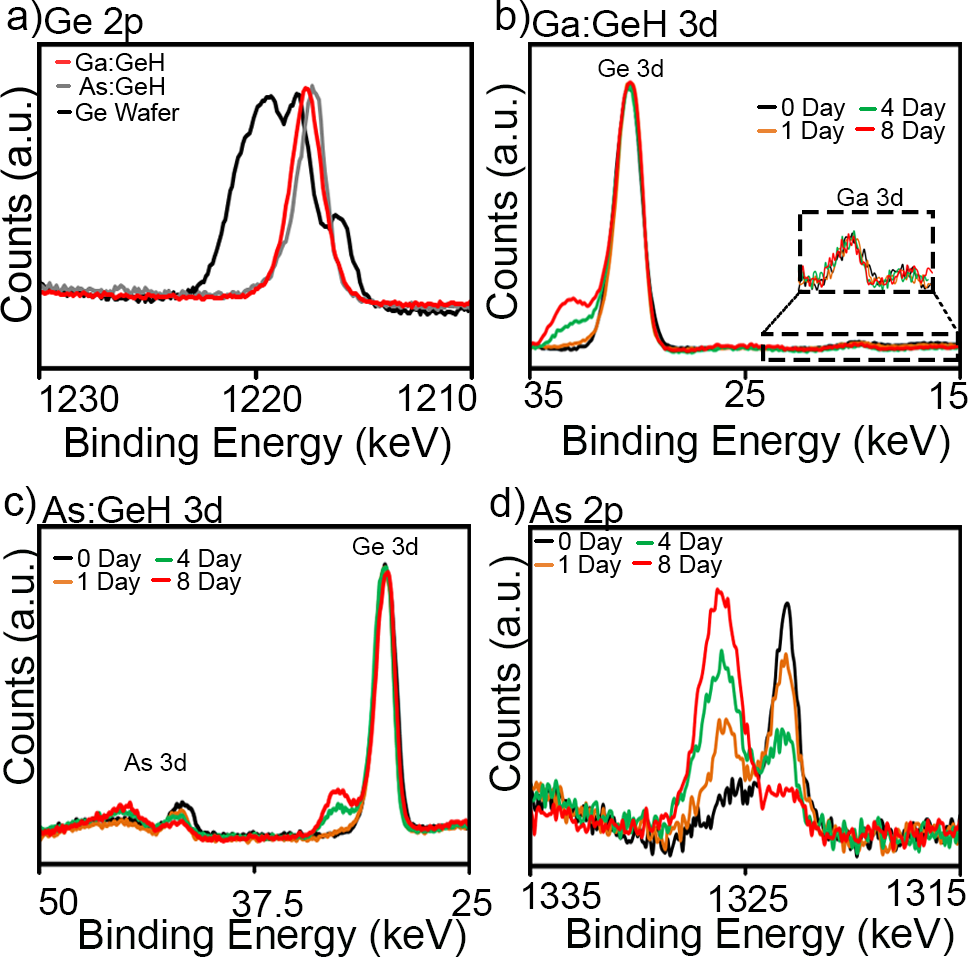
a) Ge 2p_3/2_ Ge wafer (black) with surface oxide and 2.3% Ga:GeH (red) and 1.1% As:GeH (gray) exposed for 0 days. b) Time-dependent XPS of 2.3% Ga:GeH and c,d) 1.1% As:GeH at 0 day (black), 1 day (yellow), 4 days (green), and 8 day (red).

X-ray photoelecton spectroscopy (XPS) measurements confirmed dopant retention in the lattice and elucidated the local chemical environment of the dopant (Figure 4). The Ge 2p_3/2_ peak for the 2.3% Ga:GeH and 1.1% As:GeH occur at 1217.7 and 1217.6 eV (Figure 4a), respectively, which is relatively close to the value of undoped GeH at 1217.8 eV [25]. These Ge 2p_3/2_ energies are indicative of a Ge^1+^ oxidation state. For comparison, a Ge(111) wafer having surface oxide contains Ge 2p_3/2_ peaks at 1216.3 for Ge^0^, and peaks of oxidized Ge^2+^ to Ge^4+^, which range from 1218.2 to 1220.6 eV. Figure 4b shows the Ga 3d_5/2_ and Ge 3d_5/2_ XPS spectra for the 2.3% Ga:GeH crystals after exposure to ambient conditions for 0–8 days. Immediately after synthesis (zero days of air exposure) the Ga and Ge 3d_5/2_ peaks can be fit to single peaks at 19.9 eV and 30.3 eV, respectively. These binding energies occur in the range expected for Ga^3+^ [33] and Ge^1+^ [25] oxidation states. Minimal changes are observed after one day of exposure to air. However, after four days of ambient air exposure, the XPS spectra shows the emergence of Ge 3d_5/2_ peaks at higher energies, which are indicative of surface oxidation. Fitting the higher-energy spectra shows that 83% of Ge^1+^ at the surface is not oxidized. The binding energies of the Ga 3d_5/2_ peak and of the Ga 2p_3/2_ peak that occurs at 1117.5 eV [34] do not change after exposure to ambient atmosphere. As a dopant in GeH, Ga is bonded to three more electronegative Ge atoms, and locally exists in an electron-deficient state. Consequently, minimal changes in the Ga XPS spectra would be expected if Ga:GeH were to become oxidized to form Ga_2_O_3_.

Figure 4c shows the XPS spectra for As:GeH after exposure to air for 1–8 days. Again, immediately after synthesis (0 days of air exposure) the As and Ge 3d_5/2_ peaks can be fit to single peaks at 41.8 eV and 30.0 eV, respectively. Also, in As:GeH there are only minimal changes of the observed Ge 2p_3/2_ peak after one day of exposure to air. However, surface oxidation is prevalent after four days, evident from the emergence of a peak with higher binding energy, indicative of an oxidized Ge 3d_5/2_ environment. Fitting the intensity of the peaks shows 84% of Ge remains as Ge^1+^. The similarity of the change in Ge binding energy for both As:GeH and Ga:GeH implies that the rates of oxidation of Ge in both samples are similar. In contrast to Ga:GeH, the changes in the As 2p_3/2_ binding energy (Figure 4d) indicates that significant oxidation of As occurs. The As 2p_3/2_ peak centered at 1323 eV in as-grown As:GeH starts disappearing in favor of a 1326.1 eV [35] oxidized state.

**Figure 5:**
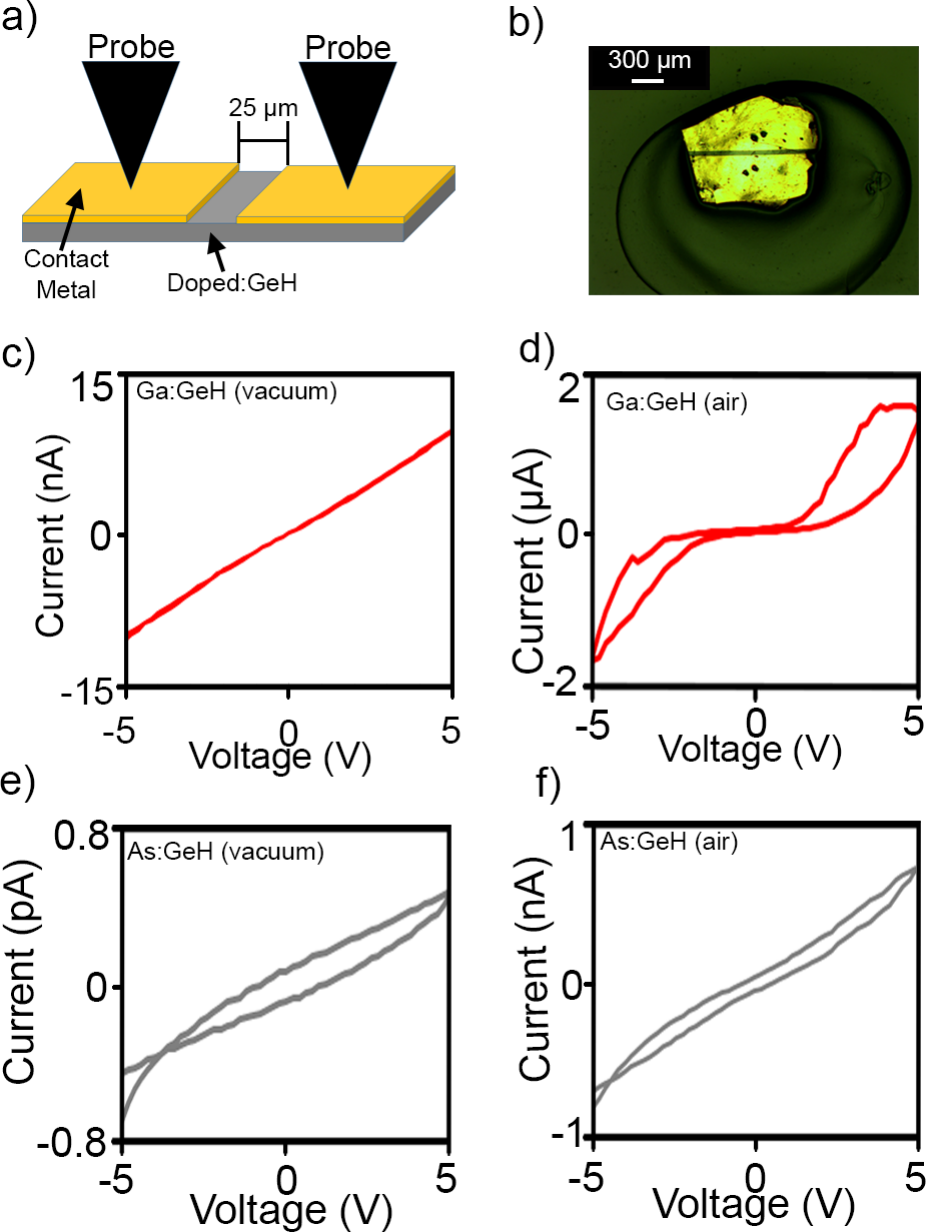
a) Diagram and b) photo of typical device for two-probe *I–V* measurements. Representative curves of 2.3% Ga:GeH (red) in c) vacuum and d) air and 1.1% As:GeH (gray) in e) vacuum and f) air.

The effect of dopants on the electronic transport of single-crystal flakes of Ga:GeH and As:GeH were measured with contacts fabricated by using a shadow mask technique (Figure 5b). Two-probe *I*–*V* measurements were carried out on single crystals with device geometries that typically featured a channel length of 25 µm, a width of 2–4 mm and a thickness of 5–20 µm (Figure 5b). After exploring numerous metals, nearly ohmic contacts (under vacuum) to Ga:GeH were observed using 100 nm Au as a contact metal. Furthermore, the highest ambient and vacuum conductivities in As:GeH were achieved when contacting with Ag (80 nm)/Au (20 nm). The fact that Au with its higher work function is needed to make ohmic contacts for Ga:GeH, compared to Ag for As:GeH, suggests that Ga and As are likely to act as p-type and n-type dopants, respectively. *I*–*V* measurements were carried out with a direct probe contact to each metal pad, and measured in a range of −5 to 5 V. 20–30 devices were fabricated for each doping concentration. Each measurement was normalized to a sheet resistance. Undoped GeH exhibited sheet resistances approaching the noise limit of the instrumentation of the order of ca. 10^15^ Ω/sq in both vacuum and under ambient conditions, similar to previous studies [29].

**Figure 6:**
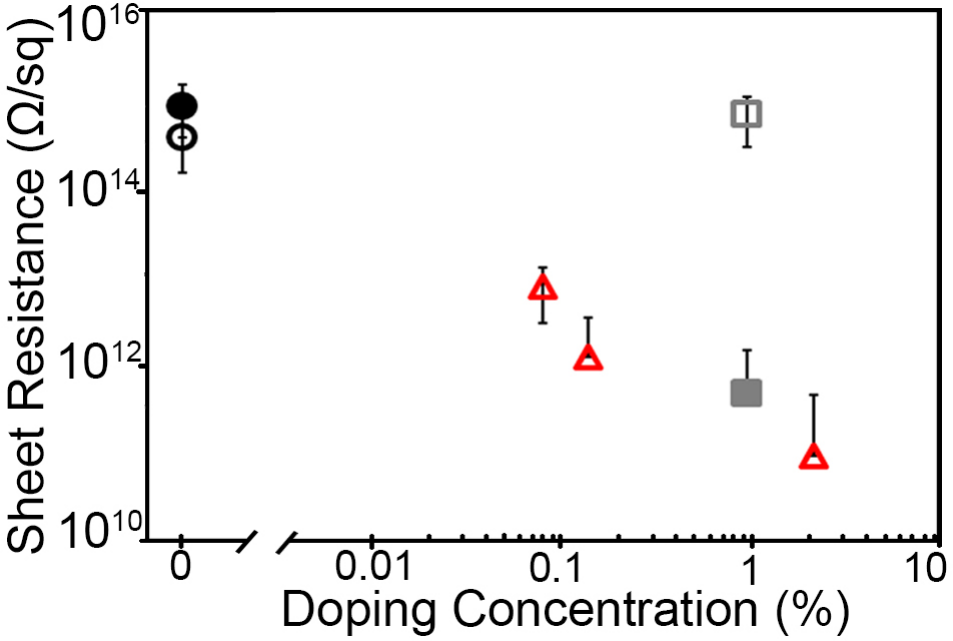
Sheet resistance of GeH (black), 1.1% As:GeH (gray), and 2.3% Ga:GeH (red) in vacuum (empty) and air (filled). Each doped data point represents 20–30 device measurements.

Figure 5c,d shows a representative *I*–*V* plot for 2.3% Ga:GeH in vacuum and air, respectively. The *I*–*V* plot for the device measured under vacuum in Figure 5c shows ohmic contact behavior, with a typical sheet resistance of 9.5 × 10^10^ Ω/sq. The *I*–*V* behavior when measured in H_2_O-containing atmosphere such as air, is highly hysteretic, non-ohmic, and with much higher current. Previously, we had shown that for P:GeH [29], H_2_O-containing atmospheres are necessary to activate the phosphorus (group 15) dopants. For Ga:GeH, the presence of atmospheric water, while significantly increasing conductivity, also introduces a significantly non-linear behavior in the *I*–*V* plots, suggesting that H_2_O plays an additional role in these gallium samples in addition to dopant activation. This also makes it difficult to extract an accurate value for sheet resistance, leading to the omission of its use as a metric for these samples. Regardless of the chemical state of the dopant atom in H_2_O atmosphere, under vacuum there is a systematic decrease of sheet resistance for Ga:GeH with increasing amounts of Ga doping. With 0.08%, 0.14%, and 2.3% of gallium doping, the sheet resistance drops to 8.4 × 10^12^, 1.3 × 10^12^, and 9.5 × 10^10^ Ω/sq, respectively (Figure 6). This means a marked improvement of more than four orders of magnitude over undoped GeH of our samples with the highest doping.

On the contrary, As:GeH does not exhibit any dopant activation under vacuum. Figure 5e shows a representative *I*–*V* plot of a 1.1% As:GeH device under vacuum. With the application of ±5 V, a current of less than 1 pA is observed, which is again at the actual noise limit of our instrumentation. However, in air, there is an increase in conductivity of at least three orders of magnitude. Figure 5f shows a representative *I*–*V* plot of As:GeH in ambient conditions. In contrast to Ga:GeH, the *I*–*V* plot is linear, with minimal hysteresis. The *I*–*V* behavior both in air and vacuum is similar to what was previously reported to occur in P:GeH, another group-15 dopant. In air, the average sheet resistance for 30 As:GeH devices was 5.0 × 10^11^ Ω/sq (Figure 6).

## Conclusion

Here we have demonstrated that gallium and arsenic can be incorporated into a precursor CaGe_2_ Zintl phase and are retained in the 2D germanium framework after the topotactic deintercalation process. These dopants do not significantly change the structure of germanane. The doped materials are stable in ambient atmosphere conditions for at least 24 h but start to oxidize after one to four days. The introduction of Ga and As to the lattice decreases the resistance under ambient conditions with large amounts of hysteresis, suggesting that the presence of water can activate these dopants. As was previously observed with P:GeH [29], As:GeH is highly resistive under vacuum, indicating the presence of water is required to activate group-15 dopants. In contrast, Ga:GeH exhibited sheet resistances in vacuum decreased by over four orders of magnitude, proportional to the amount of gallium and with minimal hysteretic behavior. This indicates that the activated state of the dopant in Ga:GeH is stable under vacuum, enabling robust electronic properties through encapsulation. Overall, this work provides a pathway to dope germanane and enable future explorations of electronic devices.

## Experimental

Single crystalline platelets of doped and undoped GeH were synthesized using methods adapted from those reported previously [25]. For undoped GeH, stoichiometric amounts of calcium (Acros, 99%) and germanium (Acros, 99.999%) were sealed in quartz tubes under vacuum of less than 60 mTorr. The sample was annealed at 950 °C for 18 h, and slowly cooled to room temperature over the course of 2–10 days. CaGe_2_ crystals were collected and placed in concentrated HCl at −40 °C for more than 8 days resulting in flakes having lateral dimensions of 5 × 5 mm. To prepare extrinsically doped CaGe_2_, elemental aluminium (Johnson Matthey Electronics 99.9%), gallium (Acros 99.9%), arsenic (Sigma 99.999%), or antimony (Strem 99%) was used to replace germanium in the initial calcium and germanium mixture. Again, these materials were sealed in quartz tubes under vacuum and annealed following the same procedures as undoped germanane. The experiments with Sb resulted in the formation of a mixture of different phases, none of which were structurally similar to any known layered CaGe_2_ polymorph. Subsequently, the single crystals of the *x*:CaGe_2_ (*x* = Al, Ga, As) were placed in −40 °C HCl for at least 8 days, until deintercalation was complete and a lack of crystalline CaGe_2_ peaks appeared in the XRD. The products were first rinsed with deionized water followed by rinsing with methanol, three times each. The crystals were collected via slow centrifugation and subsequently dried in vacuum.

The structure of doped GeH was confirmed using capilary X-ray diffraction using Cu Kα_1_ radiation (λ = 1.54 nm) on a Bruker D8 powder X-ray diffractometer. XRD was performed using with finely ground powders packed in capillaries. Raman spectroscopy was used to confirm vibrational modes using a Renishaw InVia Raman equipped with a CCD detector exciting with a 633 nm He–Ne laser. The relative elemental composition was measured using X-ray fluorescence on an Olympus X-5000 Mobile XRF System. SEM and EDX were performed using a FEI Helios Nanolab 600 dual beam focussed ion beam/scanning electron microscope. X-ray photoelectron spectroscopy was performed using a Kratos Axis ultra X-ray photoelectron spetrometer with a monochromatic aluminium X-ray gun. Samples were mounted in a glovebox and then stored in ambient atmosphere for 1, 4 and 8 days to determine the stability in air. Fourier transform infrared spectra were collected with a Perkin-Elmer frontier dual-range FIR/Mid-IR spectrometer that was loaded in an Ar-filled glovebox and using an attenuated total internal reflection (ATR) sample geometry.

Electrical properties were measured in top-contact device geometry, where metal contacts were first deposited via e-beam deposition using a shadow mask resulting in a 25 µm channel length. The contact metals used for undoped GeH and As:GeH were 80 nm/20 nm (Ag/Au). Ga:GeH device contacts were prepared using 100 nm Au. Contact materials were selected after testing with multiple metals and selecting the metal that gave the highest current and most linear *I*–*V* characteristics. Additionally, four-probe measurements indicate that the contact resistance for the 2.3% Ga:GeH is at least two orders of magnitude lower than the resistance of the material, indicating that contact resistance is negligible for these samples. All devices were stored in an Ar-filled glovebox until atmospheric measurements were carried out. Electronic measurements were conducted using a Keithley 4200-SCS attached to a Lake Shore Cryonics Inc. probe station. Two-probe current–voltage measurements were performed in both vacuum (ca. 10^−4^ mbar) and under ambient conditions in the dark.
